# Evaluation of Antibody-Dependent Fc-Mediated Viral Entry, as Compared With Neutralization, in SARS-CoV-2 Infection

**DOI:** 10.3389/fimmu.2022.901217

**Published:** 2022-05-31

**Authors:** Lindsay Wieczorek, Michelle Zemil, Mélanie Merbah, Vincent Dussupt, Erin Kavusak, Sebastian Molnar, Jonah Heller, Bradley Beckman, Suzanne Wollen-Roberts, Kristina K. Peachman, Janice M. Darden, Shelly Krebs, Morgane Rolland, Sheila A. Peel, Victoria R. Polonis

**Affiliations:** ^1^ U.S. Military HIV Research Program, Walter Reed Army Institute of Research, Silver Spring, MD, United States; ^2^ Henry M. Jackson Foundation for the Advancement of Military Medicine, Bethesda, MD, United States; ^3^ Diagnostics and Countermeasures Branch, Walter Reed Army Institute of Research, Silver Spring, MD, United States

**Keywords:** SARS-CoV-2, coronavirus – COVID-19, variant of concern, antibody dependent enhancement, neutralization, pseudovirus, Fc-mediated virus entry, spike (S) protein

## Abstract

Fc-mediated virus entry has been observed for many viruses, but the characterization of this activity in convalescent plasma against SARS-CoV-2 Variants of Concern (VOC) is undefined. In this study, we evaluated Fc-mediated viral entry (FVE) on FcγRIIa-expressing HEK293 cells in the presence of SARS-CoV-2 convalescent plasma and compared it with SARS-CoV-2 pseudovirus neutralization using ACE2-expressing HEK293 cells. The plasma were collected early in the pandemic from 39 individuals. We observed both neutralization and FVE against the infecting Washington SARS-CoV-2 strain for 31% of plasmas, neutralization, but not FVE for 61% of plasmas, and no neutralization or FVE for 8% of plasmas. Neutralization titer correlated significantly with the plasma dilution at which maximum FVE was observed, indicating Fc-mediated uptake peaked as neutralization potency waned. While total Spike-specific plasma IgG levels were similar between plasma that mediated FVE and those that did not, Spike-specific plasma IgM levels were significantly higher in plasma that did not mediate FVE. Plasma neutralization titers against the Alpha (B.1.1.7), Beta (B.1.351), Gamma (P.1) and Delta (B.1.617.2) VOC were significantly lower than titers against the Washington strain, while plasma FVE activity against the VOC was either higher or similar. This is the first report to demonstrate a functional shift in convalescent plasma antibodies from neutralizing and FVE-mediating against the earlier Washington strain, to an activity mediating only FVE and no neutralization activity against the emerging VOC, specifically the Beta (B.1.351) and Gamma (P.1) VOC. It will be important to determine the *in vivo* relevance of these findings.

## Introduction

SARS-CoV-2 is a Betacoronavirus that is the causative agent of coronavirus disease 2019 (COVID-19). COVID-19 has a broad spectrum of disease presentation; an estimated 80% of patients are asymptomatic or have mild flu-like symptoms, while 20% of patients develop severe respiratory illness (1, 2). Critical outcomes include respiratory failure, multiple organ dysfunction and shock. While determinants of disease severity and duration may be impacted by patient’s age and underlying health conditions, there is also growing evidence that the adaptive humoral immune response itself may correlate with COVID-19 disease severity. Patients with severe COVID-19 symptoms have been shown to develop earlier and higher concentrations of SARS-CoV-2 specific IgG as compared to patients with mild symptoms (3–5).

The SARS-CoV-2 spike (S) protein is the main viral protein exposed on the virion surface and is the primary target for the immune response. The S protein mediates virus entry through the human angiotensin converting enzyme 2 (ACE2) receptor and is therefore responsible for directing the host range and tissue tropism (6, 7). The coronavirus S protein is a class I fusion protein that is composed of two subunits; the S1 head subunit mediates cellular attachment, and the S2 stalk subunit mediates membrane fusion (8). Cleavage of the S protein by host proteases separates the S1 and S2 subunits, which remain non-covalently associated in a metastable, homotrimeric prefusion formation. The S1 subunit contains two major domains, the N-terminal domain (NTD) and the C-terminal domain (CTD). The CTD includes the receptor-binding domain (RBD) positioned at the top of the trimeric S (9). Binding of SARS-CoV-2 S RBD to cellular ACE2 results in infection of pneumocytes and other host cells that express ACE2.

Antibodies against the S protein have been shown to be protective against infection and disease severity (10–12). Antibodies are a critical feature of adaptive immunity that provide protection through several mechanisms, including viral neutralization or clearance (Ab dependent cellular phagocytosis, ADCP) and elimination of infected cells (Ab-dependent cellular cytotoxicity, ADCC). Potent neutralizing monoclonal antibodies (mAbs) that target the SARS-CoV-2 S RBD and NTD can inhibit viral infection by blocking receptor engagement or by interfering with the post-attachment fusion processes (10, 13, 14). Additionally, NTD neutralizing mAbs and vaccine-elicited antibodies have been shown to leverage Fc-mediated protective activities, including monocyte, neutrophil and complement engagement (10, 13, 15).

While Fc-mediated functions can be protective, plasma IgG can also facilitate infection by promoting antibody-dependent enhancement (ADE). ADE can occur when suboptimal neutralizing or non-neutralizing antibodies bind to the virus and facilitate entry in Fc receptor expressing cells (16). Viral-induced enhanced disease has been most notably reported for Dengue virus (DENV), where pre-existing, cross-reactive Abs from one DENV serotype increase infection and clinical severity of another DENV serotype or the related Zika virus (17). ADE expands the infectable host cell range and increases the cellular viral load. Binding of virus-antibody complexes to FcγRII on immune cells initiates receptor-mediated signaling for cellular activation and enhanced inflammatory cytokine expression. In vitro ADE, as defined by Fc-mediated viral entry (FVE), has been reported for many viruses, including SARS-CoV and MERS, and in some cases has been shown to impact disease severity in humans or animal models (16, 18–20).

Enhanced viral entry of infecting SARS-CoV-2 virions by convalescent plasma has been observed in vitro (21, 22) using the Washington or Wuhan viral strains from very early in the pandemic. In this study, we evaluated the impact of SARS-CoV-2 convalescent plasma antibodies on in vitro viral entry of SARS-CoV-2 pseudoviruses in ACE2 or FcγRIIa-expressing target cells. We determined plasma neutralization and FVE activity against the early SARS-CoV-2 Washington strain, as well as the variants of concern (VOC) Alpha (B.1.1.7), Beta (B.1.351), Gamma (P.1) and Delta (B.1.617.2). Additionally, we evaluated plasma antibody binding features that associate with in vitro plasma neutralization and/or FVE. Convalescent plasma FVE activity against the infecting strain was observed in 31% of participants and was significantly associated with lower S-specific IgM binding. The plasma dilution at which maximum FVE activity was observed correlated significantly with neutralization titer, indicating that as antibody concentration decreases and neutralization wanes, Fc-mediated uptake peaks. Plasma neutralization of VOC was significantly lower compared to neutralization of the Washington strain, while FVE against the VOC was higher or similar. These data indicate that increasing neutralization-resistance of VOC may facilitate an uptick in viral entry in the absence of neutralizing plasma activity.

## Results

### Production and Characterization of FcγRIIa- and ACE2-Expressing Target Cell Lines

**Figure 1:**
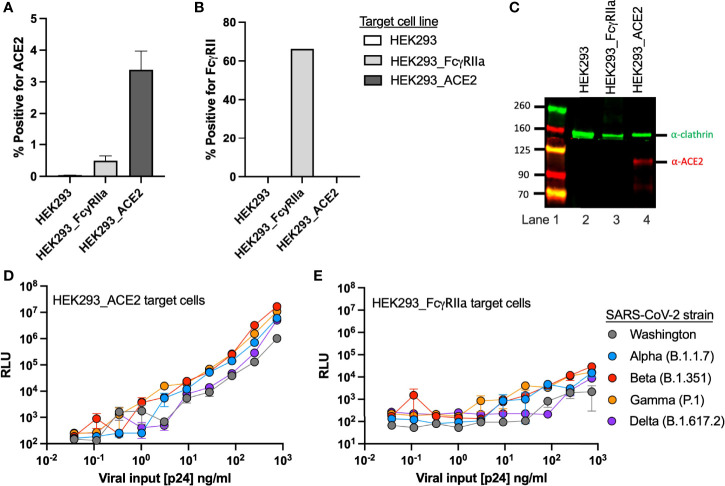
Target cell line characterization. Surface expression of **(A)** ACE2 and **(B)** FcγRII receptors on HEK293, HEK293_ACE2 and HEK293_ FcγRIIa target cell lines detected by flow cytometry. **(C)** Target cell expression of ACE2 detected by western blot. The blot shows a molecular weight marker (lane 1) along with ACE2 (red) and clathrin (green) detection for the HEK293 (lane 2), HEK293_ FcγRIIa (lane 3), and HEK293_ACE2 (lane 4) cell lines. Infectivity of the SARS-CoV-2 PSV strains on the **(D)** HEK293_ACE2 and **(E)** HEK293_ FcγRIIa target cell lines.

Given the previous observations of FcγRIIa-mediated entry of SARS-CoV (18), we transduced HEK293 cells with an FcγRIIa expression vector to produce a stable FcγRIIa expressing cell line, and then utilized it to evaluate FVE of SARS-CoV-2 pseudoviruses (PSVs). For comparison with FVE, we utilized a commercially available HEK293 ACE2-expressing cell line to evaluate ACE2-mediated PSV entry and neutralization. Cell surface expression of FcγRII and ACE2 receptors was evaluated by flow cytometry for both cell lines and the parental HEK293 cells (**Figures 1A, B**). We observed low ACE2 surface expression (3.4% positive) for the HEK293_ACE2 cell line, while the FcγRIIa-expressing cell line was about 65% positive for FcγRII. We confirmed the former expresses ACE2 by western blot (**Figure 1C**). The 110 kDa ACE2 protein and an ˜80 kDa cleavage product (lane 4, red infrared (IR) bands) were detectable for only the HEK293_ACE2 cell line. Clathrin, a housekeeping gene, was used as a control for each cell line (green IR bands).

We then evaluated susceptibility of the target cell lines to viral entry by five SARS-CoV-2 S variants, including the original US variant Washington, and the Alpha (B.1.1.7), Beta (B.1.351), Gamma (P.1) and Delta (B.1.617.2) VOC. To establish baseline viral entry and to compare infectivity of these PSV strains, we performed viral titrations on the HEK293_ACE2 and HEK293_FcγRIIa cell lines (**Figures 1D, E**). Viral input was quantified and standardized by determining the pseudotyped HIV p24 capsid concentration of each viral stock. For both target cell lines we observed greater infectivity with the VOC as compared to the Washington strain (grey), which was approximately one log less infectious than the Beta variant (red).

### FcγRIIa and ACE2-Mediated Entry of SARS-CoV-2 Pseudovirus in the Presence of Convalescent Plasma

**Figure 2:**
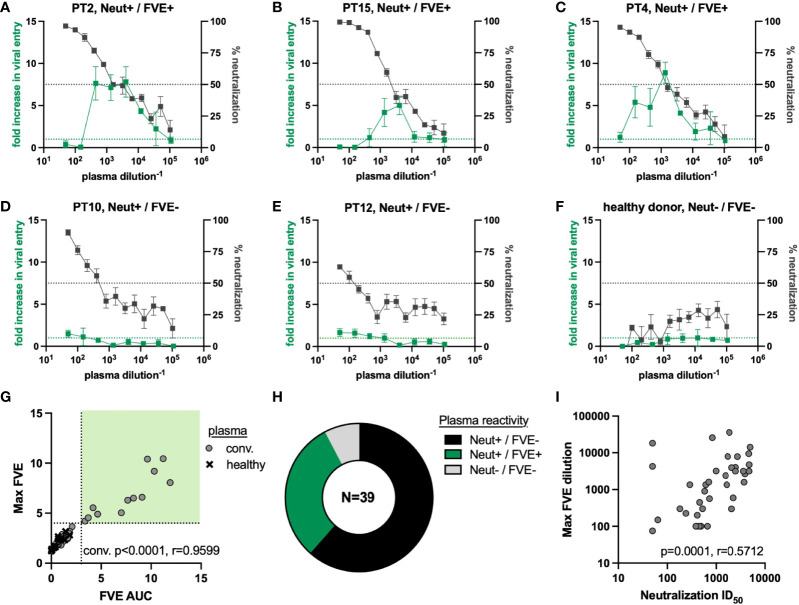
SARS-CoV-2 convalescent plasma reactivity against the SARS-CoV-2 Washington strain PSV. **(A–F)** Plasma titrations on the HEK293_ACE2 (right axis in gray, % neutralization) and HEK293_ FcγRIIa (left axis in green, fold increase in viral entry in the presence of plasma) target cell lines. Representative titers show relationship between FVE and neutralization for **(A–C)** individuals that mediate FVE and neutralization, **(D)** and **(E)** individuals that only mediate neutralization, and **(F)** healthy donor plasma. **(G)** Correlation between the FVE endpoints of max FVE and FVE AUC used to define plasma as FVE mediating (upper right quadrant). Convalescent plasma samples are shown as circles and pre-pandemic, healthy human plasma samples are shown as ‘X’ symbols. **(H)** Percentages of plasma mediating FVE and/or neutralization against the SARS-CoV-2 Washington strain PSV. **(I)** Spearman correlation between the FVE endpoint of plasma dilution at which max FVE was observed and neutralization ID_50_.

We performed viral entry assays in the presence of convalescent (n=39) or healthy donor (n=16) plasma to observe the impact of plasma antibody on FcγRIIa or ACE2-mediated entry of the SARS-CoV-2 Washington strain PSV. All convalescent patients reported mild symptoms; plasma was not available from severe infection or asymptomatic individuals. Fold increase in viral entry in the presence of plasma antibodies, as compared to virus only, on HEK293_FcγRIIa target cells (green lines) and % viral neutralization on HEK293_ACE2 target cells (black lines) is shown for five representative convalescent plasma samples (**Figures 2A–E**) and one representative healthy donor plasma (**Figure 2F**). The black dotted line indicates 50% neutralization, and the green dotted line indicates the baseline level of viral entry in the absence of plasma in the HEK293_FcγRIIa cells. Three representative samples showed a 5 to 10-fold maximum increase in entry in the presence of plasma (FVE+; PTs 2, 4, and 15), while two plasmas showed minimal FcγRIIa-mediated increase in viral entry (FVE-; PTs 10 and 12). All five represented samples showed >50% neutralization for at least 2 dilutions. Three endpoint characteristics were determined to define FVE and quantify FcR-mediated entry into HEK293_FcγRIIa cells in the presence of antibody. These endpoint characteristics included area under the curve >1 (FVE AUC), maximum fold increase in viral entry (max FVE), and the plasma dilution at which maximum viral entry was observed. Plasma-mediated neutralization on HEK293_ACE2 cells was reported as the 50% inhibitory dilution (ID_50_).

We observed a significant relationship between FVE AUC and max FVE for the convalescent plasma, as expected (**Figure 2G**, p<0.0001, r=0.9599). We compared convalescent plasma to healthy donor plasma activities to define viral entry greater than 4-fold above the level of viral entry seen without plasma, and an AUC greater than 3 (**Figure 2G**; FVE+ quadrant shown in light green). By these criteria, all healthy donor plasma were FVE- and FVE was maintained over multiple dilutions in samples defined as FVE+. Using these FVE cutoff criteria, we observed that convalescent plasma from 12 individuals (31%, green) mediated FVE and neutralization, 24 individuals (61%, black) mediated neutralization but not FVE, and 3 individuals (8%, grey) didn’t mediate FVE or neutralization of the SARS-CoV-2 Washington strain (**Figure 2H**). Individuals in these functional plasma activity groups were not distinguished by available clinical or demographic data. Plasma from all healthy donors did not mediate FVE or neutralization (**Figure 2F** and data not shown).

No correlation was observed between FVE AUC or max FVE and the dilution at which the maximum viral entry was observed (p=0.2348. r=-0.1948, and p=0.3172, r=-0.1644 respectively; data not shown). However, we observed a significant correlation between the plasma dilution at which the max FVE was observed and the plasma neutralization ID_50_ value (**Figure 2I**; p=0.0001, r=0.5712), indicating an intersection between the decline of plasma antibody neutralizing capacity and the peak FVE activity.

### FcγRIIa and ACE2-Mediated Entry of SARS-CoV-2 VOC Pseudoviruses in the Presence of Convalescent Plasma

**Figure 3:**
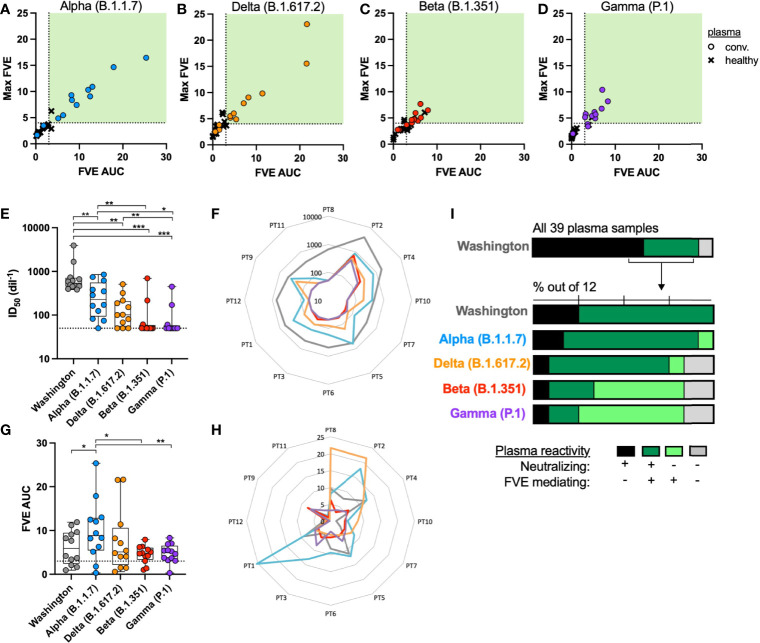
SARS-CoV-2 convalescent plasma reactivity against the SARS-CoV-2 VOC PSVs. Correlation between the FVE endpoints of max FVE and FVE AUC using the **(A)** Alpha (B.1.1.7), **(B)** Delta (B.1.617.2), **(C)** Beta (B.1.351) and **(D)** Gamma (P.1) VOC; convalescent plasma samples are shown as circles and pre-pandemic, healthy human plasma samples are shown as ‘X’s. Differences in plasma **(E)** neutralization and **(G)** FVE across all tested SARS-CoV-2 VOC (Wilcoxon test, *p < 0.05, **p < 0.005, ***p < 0.0005). In **(F, H)** the radar plots display the neutralization ID_50_s **(F)** or FVE fold-increase in entry **(H)** for the five tested VOC, with the neutralization or FVE values increasing from the center outward, as indicated by the concentric light grey lines. The participant (PT) identifiers are indicated at the perimeter of the plot and values for each strain are plotted in colored lines, maintaining the same color code as indicated in the scatter plots in **(E, G)**. **(I)** Percentages of plasma mediating FVE alone (light green), FVE and neutralization (dark green), neutralization alone (black) or neither activity (grey) against the five tested SARS-CoV-2 VOC.

We next analyzed a subset of 12 convalescent plasma samples (3 plasmas mediated neuralization but not FVE, and 9 plasmas mediated both neuralization and FVE against the Washington strain) and 16 healthy plasma samples to evaluate the impact of plasma antibody on FcR or ACE2-mediated entry of the SARS-CoV-2 Alpha (B.1.1.7), Beta (B.1.351), Gamma (P.1) and Delta (B.1.617.2) VOC PSVs. The VOC are shown in consistent colors throughout the figures: Alpha = blue, Delta = orange, Beta = red, and Gamma = purple. The same definition of FVE was used to compare viral entry between convalescent and healthy donor plasma for each VOC (**Figures 3A–D**).

A significant reduction in plasma neutralization was observed against all VOC, as compared to the Washington strain; results are shown against each strain in aggregate by all plasmas in **Figure 3E**. While all 12 of the convalescent plasma samples neutralized the Washington strain, only 3 or 4 convalescent plasma samples neutralized the Gamma (P.1) or Beta (B.1.351) VOC, respectively. In the radar plot shown in **Figure 3F**, the color-coded lines indicate the ID_50_ for each convalescent participant (PT) plasma against a given strain. PT plasma number is indicated at the perimeter, and the closer the point is to the perimeter, the greater the plasma neutralization is for that PT. The grey line indicates the higher level of neutralization of the Washington strain by each PT, as compared to the lower ID_50_s for all other variants, likely due to the fact these individuals were infected with the Washington strain. There was no significant correlation between plasma neutralization activity against the Washington strain and any of the VOC (data not shown). Conversely, we observed a significant increase in plasma-mediated viral entry against the Alpha (B.1.1.7) variant, while the FVE of the other VOC was comparable to that of the Washington strain (**Figure 3G**). Notably, **Figure 3H** demonstrates that two PT samples mediated higher FVE for the Alpha and/or Delta variants as compared to the Washington variant.

Within this subset of 12 convalescent plasma samples selected for analysis against the VOC, there was an expansion of the individuals that demonstrated FVE, but no neutralization, as can be seen in the lighter green bars in **Figure 3I**. A shift in plasma activity from antibody-dependent viral entry and neutralization against the Washington strain to antibody-dependent viral entry without neutralization against the Beta (B.1.351) and Gamma (P.1) VOC was specifically observed (**Figure 3I**).

### Convalescent Plasma Antibody Specificity and FcR Binding

**Figure 4:**
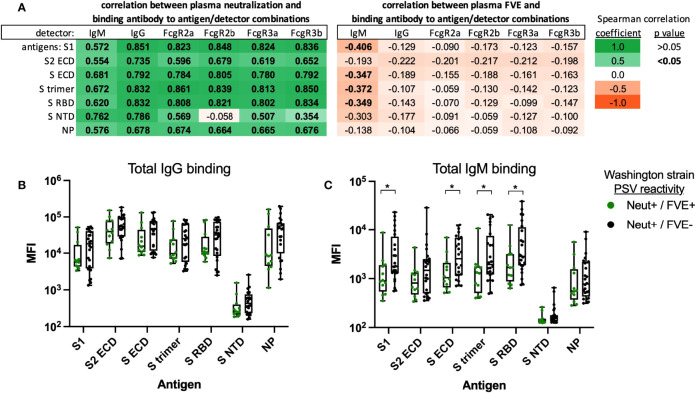
Correlation between convalescent plasma binding antibody responses and plasma neutralization or FVE activity against the SARS-CoV-2 Washington strain. Binding antibody responses were measured by Luminex multiplex assay to determine the antibody specificity and Fc reactivity of convalescent plasma IgG. The combination of antibody antigen and Fc binding is shown for Washington strain antigens (shown in rows) and Ig or Fc detectors (shown in columns), in correlation with plasma (**A**-left) neutralization or (**A**-right) FVE. Spearman correlation coefficients are shown to indicate strength and direction of the trend; trends with significant p values are shown in bold. The magnitude of antigen-specific plasma **(B)** IgG and **(C)** IgM are shown for plasma that mediated neutralization and FVE (Neut+/FVE+, green) or neutralization and not FVE (Neut+/FVE-, black). Significant differences determined by Mann-Whitney U test are indicated; *p < 0.05.

Plasma binding antibody responses in all 39 convalescent plasma were evaluated by Luminex multiplex assay using Washington strain antigens to determine if plasma antibody specificity, subclass or Fc reactivity impacted plasma neutralization and/or FVE. We evaluated the correlation between convalescent plasma neutralization (**Figure 4A**, left) or plasma FVE (**Figure 4A**, right) against the Washington strain, with the magnitude of antigen (shown in rows) and Fc-specific (shown in columns) binding antibody responses. Analysis of Fc-reactivity included characterization of Ig isotype, including IgM and IgG, or binding to common Fcγ receptors, including FcγRIIa, FcγRIIb, FcγRIIIa and FcγRIIIb. The magnitude of binding antibodies directed to SARS-CoV-2 antigens correlated directly with plasma neutralization for all Fc detectors, except for FcγRIIb-specific binding to S NTD antigen (p=0.7385, r -0.058; peach box in **Figure 4A** left Neutralization panel). Interestingly, significant inverse correlations were observed between plasma FVE AUC and the magnitude of IgM binding antibodies to S1, S ECD, S trimer and S RBD antigens only (p=0.014 to p=0.038, shown in bold text). The magnitude of antigen specific binding activity for total IgG or total IgM was compared between individuals that mediated neutralization and FVE versus individuals that mediated neutralization but not FVE against the Washington strain (**Figures 4B, C**, respectively). Total IgG binding was similar between groups. We observed a higher magnitude of S protein specific IgM responses for individuals that did not mediate FVE as compared to individuals with FVE; significant differences were observed for S1 (p=0.0219), S ECD (p=0.0317), S trimer (p=0.0411) and S RBD (p=0.0495) antigens, as indicated. We further compared time between participant’s onset of symptoms and plasma collection as it may impact antigen-specific IgG and IgM levels and observed no differences between groups (Neut+/FVE+: mean 32 days, range 22 – 41 days; Neut+/FVE-: mean 30 days, range 22 – 47 days).

**Figure 5:**
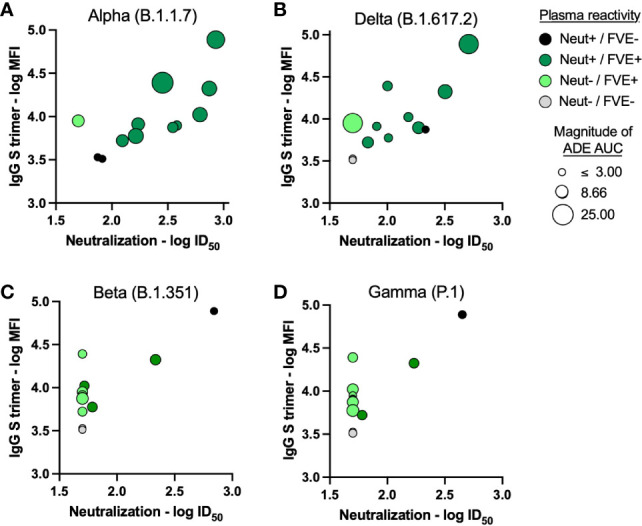
Convalescent plasma binding antibody responses in relation to plasma FVE and/or neutralization activity of SARS-CoV-2 VOC. Plasma IgG binding antibody responses to the Washington strain S trimer antigen were correlated with plasma neutralization of the **(A)** Alpha (B.1.1.7), **(B)** Delta (B.1.617.2), **(C)** Beta (B.1.351) and **(D)** Gamma (P.1) VOC. Convalescent plasma neutralization (Neut) and FVE against the respective VOC is indicated as the color of the symbol; black = Neut +/FVE -, dark green = Neut +/FVE +, light green = Neut -/FVE +, and gray = Neut -/FVE -. The size of the symbol reflects the magnitude of FVE AUC, the larger the circle the higher the observed plasma FVE.

Additionally, for the 12 convalescent plasma samples tested, we evaluated the correlation between neutralization or FVE against the VOC and antibody characteristics (binding to Washington strain antigens and Ig or Fc receptor engagement; **Table S1** and **Figures 5A–D**). We observed significant direct correlations between plasma neutralization of all VOC and multiple S antigen binding antibody responses. Plasma FVE responses also correlated significantly with S antigen binding antibody responses for the Alpha (B.1.1.7) and Delta (B.1.617.2) VOC (**Figures 5A, B**, **Table S1**), but not for the Beta (B.1.351) and Gamma (P.1) VOC (**Figures 5C, D**, **Table S1**). The relationship between plasma FVE, neutralization, and IgG binding to the Washington strain S trimer, shown in **Figure 5**, demonstrates similar trends between the Alpha (B.1.1.7) and Delta (B.1.617.2) VOC, and separately the (B.1.351) and Gamma (P.1) VOC.

As we had previously observed an inverse correlation between plasma binding antibody titers and FVE against the Washington strain for all 39 patients, we re-evaluated this relationship for this subset of 12 individuals. For this subset of patients, which primarily included patients that mediated FVE, we observe a positive correlation between binding antibody titers and FVE of the Washington strain, like what was observed for the VOC (**Table S1**, bottom). Interestingly, for this subset of patients, we observe significant direct correlations between FVE and binding antibody responses to only the S trimer and S2 ECD. However, only 12 individuals were included in this analysis and further work is needed to clarify these trends.

## Discussion

Over 513 million COVID-19 cases have been confirmed worldwide, as of 1 May 2022. While pulmonary impairment is the most common symptom of infection, concurrent extra-pulmonary manifestations and post-COVID conditions are also prevalent and diverse (23). Due to the recency of the SARS-CoV-2 pandemic, the long-term consequences of COVID-19 infection won’t be fully characterized for some time.

Persistence of symptoms beyond the initial SARS-CoV-2 infection (4 or more weeks) implicates the adaptive immune response in propagating or exacerbating long-term sequelae (24). In this study, we evaluated humoral immune responses from convalescent individuals to determine the impact of their plasma antibodies on inhibiting or mediating SARS-CoV-2 entry in ACE2- or FcR-expressing cells, respectively. We determined that convalescent plasma antibodies could both inhibit and mediate viral entry of the infecting Washington SARS-CoV-2 strain. Plasma FVE against the Washington strain was observed for 31% of convalescent individuals and was detected only in the presence of neutralizing antibodies. For these individuals, viral entry and neutralization activities correlated inversely across multiple plasma dilutions, in that FVE activity peaked when neutralizing activity was declining. Additionally, significantly lower S protein specific plasma IgM was observed in individuals that mediated FVE, indicating IgM may potentially compete with FVE-mediating S-specific IgG for S antigen binding. Previous reports have shown the protective effect of S-specific IgM (25, 26). Infected individuals that developed earlier and higher IgM responses recovered more quickly, while higher IgG levels during recovery correlated with disease severity (27, 28).

Extrinsic ADE can occur through FcR endocytosis of IgG and viral immune complexes. In vitro ADE has been documented for multiple viruses, including HIV (29), influenza (30), Ebola (31) and flaviviruses (32–34). ADE was first and most notably described for DENV (35, 36); pre-existing antibodies can enhance infectivity and virulence of a secondary infection, increasing likelihood of Dengue hemorrhagic fever and dengue shock syndrome (37, 38). ADE occurs when non-neutralizing or sub-neutralizing concentrations of antibody bind to viral antigens without inhibiting or clearing infection. This cross-reactive but reduced-affinity antibody binding has been observed across DENV serotypes and between flaviviruses (39–41). In this study, we similarly observed Fc-mediated entry coinciding with declining or sub-neutralizing plasma antibody dilutions but have refrained from using the term ADE in reference to this activity as it is unclear whether true “enhancement” is occurring in this in vitro setting. Thus, we have preferred to define the activity simply as FcγR-mediated virus entry, or FVE. The role of IgG Fc receptors in potential ADE of SARS-CoV-2 infection is still unclear. However, as neutralization-resistant SARS-CoV-2 VOC emerge, further understanding of plasma antibody cross-reactivity and potential ADE are needed (42).

Several mechanisms of coronavirus FVE have been reported. RBD mAbs have been shown to trigger conformational changes in MERS or SARS-CoV S, facilitating entry into Fc receptor expressing cells through receptor mimicry (18, 43). Additionally, infection enhancing SARS-CoV-2 NTD mAbs, isolated from a patient with severe COVID-19, have been shown to induce conformational changes in S that promote ACE2 binding and fusion (44). In this study, we evaluated Fc-mediated viral entry utilizing FcγRIIa, as this Fc receptor was previously shown to mediate viral entry of SARS-CoV (18). Another study recently observed FcγRIIb-mediated enhancement of viral infection with RBD-specific SARS-CoV-2 neutralizing antibodies, while NTD-specific non-neutralizing antibodies were shown to mediate enhancement through a FcγR-independent mechanism (45). In this work, these infection-enhancing antibodies were protective in vivo when infused in monkeys and mice, however lung inflammation was observed in a small number of infused animals, as compared to untreated infected controls (45). Adding further layers of complexity to the potential FVE mechanisms, alternative receptors have been demonstrated to mediate or facilitate SARS-CoV-2 entry. Cellular molecules, including KREMEN1, ASGR1, and CD147 have been shown to mediate viral uptake (46, 47), while infection enhancing molecules, such as the phosphatidylserine receptor, facilitate viral internalization in an ACE2-dependent manner (48, 49). These studies demonstrate the diverse and complex cellular entry mechanisms of SARS-CoV-2 (50). Further analysis is needed to explore the potential mechanism of convalescent plasma antibody FVE observed in this study.

Here we evaluated the impact of convalescent plasma from Washington strain SARS-CoV-2 infection on viral entry of SARS-CoV-2 VOC, including Alpha (B.1.1.7), Beta (B.1.351), Gamma (P.1) and Delta (B.1.617.2). Convalescent plasma had reduced neutralization potency against all tested VOC; however, the reduction was most notable for the Beta (B.1.351) and Gamma (P.1) VOC. This is consistent with previous reports showing increased neutralization resistance of Beta (B.1.351) and Gamma (P.1) as compared to other variants, due to the shared E484K mutation in RBD (51–54). Amino acid mutations, S characteristics and monoclonal antibody neutralization sensitivity are also more similar between the Beta (B.1.351) and Gamma (P.1), as compared to Alpha (B.1.1.7) or Delta (B.1.617.2) VOC (51, 55). Similarly, we observe consistent patterns in plasma reactivity against the Beta (B.1.351) and Gamma (P.1) versus the Alpha (B.1.1.7) and Delta (B.1.617.2) VOC. FVE against the VOC was similar to or higher than the Washington strain. Thus, when plasma neutralization capacity declined significantly, plasma antibodies were found to mediate viral entry in the absence of neutralization activity. Further studies are needed to evaluate the impact of convalescent antibodies from currently circulating SARS-CoV-2 infections on FVE of emerging SARS-CoV-2 VOC that may be more neutralization-resistant.

The recently emerged Omicron (B.1.1.529) VOC contains a significantly larger number of mutations that result in neutralization resistance against many therapeutic and convalescent plasma antibodies (56, 57). Increased transmissibility of VOC has also been reported; Alpha (B.1.1.7) VOC was determined to be 43-90% more transmissible than the Washington strain, Delta (B.1.617.2) VOC was determined to be 60% more transmissible than Alpha (B.1.1.7) VOC, and Omicron (B.1.1.529) VOC was determined to be 100% more transmissible than Delta (B.1.617.2) VOC (57–59). Further, population-level analysis has identified a significant increase in reinfection risk associated with Omicron (B.1.1.529), but not Beta (B.1.351) or Delta (B.1.617.2) VOC (60). The implications of diminished reactivity of pre-existing antibodies in Omicron (B.1.1.529) reinfection have yet to be determined. However, while Omicron (B.1.1.529) is highly transmissible, disease severity of infection or reinfection appear to be diminished, with a significantly reduced risk of COVID-19 hospitalization as compared to Delta (B.1.617.2) VOC infection (61, 62).

The balance of antibody functions in vivo is complicated and cannot be reduced to functional activity observed in isolation in vitro. Neutralization and Fc-mediated antibody functions have a demonstrated role in prevention and control of SARS-CoV-2 (54, 63–65). A single antibody can have multifunctional capacity (66) by mediating both neutralization and ADCC, and similarly could mediate both ADCC and FVE, depending on environmental conditions. Passive immunotherapies have been developed for treatment and protection against emerging SARS-CoV-2 variants, recently reviewed in detail (67). Among those treatments, convalescent plasma therapies have been evaluated with mixed outcome, but are generally determined to be safe. Convalescent plasma therapy treatment effectiveness is impacted by the quality, titer and timing of administered antibodies (68–71). Qualities of the humoral immune response to SARS-CoV-2 have been shown to vary with disease severity (72–76). Patients with severe COVID-19 have unique serological signatures, including afucosylated Fc glycans which have enhanced interactions with FcγRIIIa and result in proinflammatory activity (73, 75). Our study further demonstrates varying levels of convalescent plasma antibody Fc-engagement as determined by FVE. However, we have yet to explore the potential of this antibody Fc-engagement to result in protective Fc-mediated antibody functions, such as ADCC or ADCP. Further, convalescent antibody activities have been shown to differentially wane over time, with neutralization potency declining more rapidly than Fc-mediated ADCC and ADCP (77). The dynamic nature of the SARS-CoV-2 humoral immune response and antibody functions may shift over time in the context of this evolving COVID-19 pandemic.

These experiments employed cell line models transduced to express the ACE2 or FcγRIIa receptors, and therefore receptor expression may vary from in vivo target cells. The antibody functions against SARS-CoV-2 described herein have not yet been well characterized using primary viral isolates or using lung cells or macrophages. The impact of antibody on viral entry in primary cells co-expressing ACE2 and FcγRIIa receptors is unknown (as illustrated in **Figure S1** as a graphical abstract). Viral entry and cytokine expression, which was not measured in this study, may vary between cell types and environmental conditions. The downstream cellular consequences of FVE observed in this study were thus not analyzed as they may not be physiologically relevant and could therefore lead to misleading conclusions. Studies using the challenging primary cell culture systems and wild type virus should shed further light on the in vivo importance of the functional activities observed in this study. While neither medical encounter, nor follow up data were available for the individual samples accessible for this study, analysis of longitudinal plasma samples from patients with varying clinical severity, and/or known reinfection status will help elucidate the relationship between in vitro Fc-mediated viral entry versus neutralization and in vivo disease progression. Despite these limitations, the data presented here suggest a relationship between S protein antibody binding titers and antibody functions that mediate virus neutralization or Fc-mediated virus entry. These observations warrant further investigation using well characterized, curated samples and primary viral and cell systems to further elucidate the impact of specific antibody functions on the pathogenesis of SARS-CoV-2 and COVID-19 disease.

## Materials and Methods

### SARS-CoV-2 Convalescent Plasma

Convalescent plasma were purchased from Seracare Life Sciences (Milford, MA) and BEI Resources (Manassas, VA) and were collected from individuals infected between February and April 2020 in the SARS-CoV-2 pandemic. Healthy donor plasma samples were from the WRAIR IRB-approved RV229 protocol and were collected from individuals prior to the SARS-CoV-2 pandemic.

### Lentiviral Particle Transduction and Culture of Cell Lines

Lentiviral particles were produced by co-transfecting HEK293T/17 cells (ATCC, Mannassas, VA) with a Lenti-Pac HIV expression packaging kit (GeneCopoeia, Rockville, MD) and an ORF expression clone for FCGR2A (GenBank Accession: NM_021642.3) with neomycin resistance (GeneCopoeia Rockville, MD) according to the manufacturer’s instructions. Lentiviral particle p24 concentration was determined by HIV-1 p24 antigen capture kit (Advanced Bioscience Laboratories, Rockville, MD) and HEK293 cells (ATCC, Mannassas, VA) were transduced with an MOI of 10. FcγRIIa expressing cells were selected for using 1 mg/mL of G418 antibiotic in the growth medium. ACE2-expressing HEK293 cells were commercially obtained (Integral Molecular, Philadelphia, PA) and cultured with 1 µg/mL of puromycin in the growth medium.

### Characterization of Target Cells by Flow Cytometry

Cells were detached using non-enzymatic cell dissociation buffer (Millipore Sigma, Burlington, MA) and 2.5 x 10 (5) cells per well were added to a 96-well plate. Cells were washed with PBS and then stained with 50 µL of LIVE/DEAD fixable violet stain kit (Thermo Fisher Scientific, Waltham, MA) according to the manufacturer’s recommendations. After washing off excess viability stain, the cells were stained extracellularly using antibodies specific for ACE2 (R&D Systems, Minneapolis, MN) or FcγRII (BD Biosciences, Franklin Lakes, NJ) for 30 mins at 4°C. The cells were then washed and fixed with 2% formaldehyde (Tousimis, Rockville, MD). Data was recorded using the Becton FACSymphony (BD Biosciences, Franklin Lakes, NJ) and post-acquisition analysis was performed with FlowJo 10.7.1 (FlowJo, Ashland, OR).

### Characterization of Target Cells by Western Blot

Cells were lysed, treated with Mammalian Protein Extraction Reagent (Thermo Fisher Scientific, Waltham, MA) containing protease inhibitors (Roche, Basel, Switzerland), including phenylmethylsulfonyl fluoride (Sigma-Aldric, St. Louis, MO) and shaken at 4°C. Extracts were centrifuged at 14,000 G at 4°C for 10 minutes, then supernatants were collected, and protein concentrations were determined using Pierce bicinchoninic acid (BCA) protein assay (Thermo Fisher Scientific, Waltham, MA). Lysates were normalized for total protein concentrations and electrophoresed along with a dual-color pre-stained protein ladder (LI-COR Biosciences, Lincoln, NE) in a 4-20% SDS-PAGE gel (Bio-Rad Laboratories, Hercules, CA). Proteins were transferred onto a nitrocellulose membrane (LI-COR Biosciences, Lincoln, NE) and blocked overnight at 4°C. The blot was probed from 150–260 kDa with anti-clathrin (Abcam, Cambridge, UK) and from 45–150 kDa with anti-ACE2 (Prosci, Poway, CA) for 2.5 hours at room temperature while rocking. The membrane was washed, then incubated with goat anti-rabbit IRDye 800 for the green channel between 150 – 260 kDa and IRDye 680 (LI-COR Biosciences, Lincoln, NE) for the red channel between 45 – 150 Kda for 45 minutes at room temperature while rocking. The membrane was washed and scanned using the Odyssey 9120 infrared imager system (LI-COR Biosciences). The images were analyzed with the Odyssey infrared imager system (LI-COR Biosciences).

### SARS-CoV-2 Pseudovirus Production and Infectivity Assay

SARS-CoV-2 pseudovirus (PSV) were produced by co-transfection of HEK293T/17 cells with a SARS-CoV-2 S plasmid (pcDNA3.1) and an HIV-1 NL4-3 luciferase reporter plasmid (pNL4-3.Luc.R-E-, NIH HIV Reagent Program, Manassas, VA). The S expression plasmid sequence was derived from the Wuhan Hu-1 strain (GenBank # NC_045512), which is also identical to the IL1/2020 and WA1/2020 strains, and was codon optimized and modified to remove the last 18 amino acids of the cytoplasmic tail to improve S incorporation into the PSV, thereby enhancing infectivity. S expression plasmids for SARS-CoV-2 VOC were produced similarly and included S sequences for Alpha (B.1.1.7), Beta (B.1.351), Gamma (P.1), and Delta (B.1.167). Concentration of p24 in PSV stocks was determined by HIV-1 p24 antigen capture kit (Advanced Bioscience Laboratories, Rockville, MD) according to the manufacturer’s protocol. To determine infectivity, PSVs were serially diluted and 25 μl/well was added to a white 96-well plate with an equal volume of growth medium. Target cells were added to each well (40,000 cells/well) and incubated at 37°C for an additional 48 hr. Bright-Glo Luciferase Assay System substrate was added and Relative Light Units (RLUs) were measured using the EnVision Multimode Plate Reader (Perkin Elmer, Waltham, MA).

### SARS-CoV-2 Pseudovirus Neutralization and FVE Assays

Test plasma were diluted 1:25 in growth medium, serially diluted, then 25 μl/well was added to a white 96-well plate. An equal volume of diluted SARS-CoV-2 PSV was added to each well and plates were incubated for 1 hr. at 37°C. HEK293 target cells expressing ACE2 or FcγRIIa were added to each well (40,000 cells/well) for neutralization or FVE assays, respectively. Plates were incubated for 48 hr. at 37°C. Then Bright-Glo Luciferase Assay System substrate (Promega, Madison, WI) was added and RLUs were measured using the EnVision Multimode Plate Reader (Perkin Elmer, Waltham, MA). Neutralization dose–response curves were fitted by nonlinear regression and final titers were reported as the reciprocal of the dilution of plasma necessary to inhibit 50% of viral entry (ID_50_). FVE was calculated as the fold increase in viral entry in the presence of plasma; curves were analyzed using GraphPad Prism to determine the area under the curve >1 (FVE AUC), maximum fold increase in viral entry (max FVE), and the plasma dilution at which maximum viral entry was observed.

### SARS-CoV-2 Antigens

Seven SARS-CoV-2 antigens were used to profile antibody responses, including S extra-cellular domain (S ECD), which comprises the subunit 1 (S1) and 2 (S2) (S1+S2 ECD), S1, S2, the nucleoprotein (NP) (Sino Biological,; 40589-V08B1, 40591-V08B1, 40590-V08B and 40588-V08B), the S Receptor Binding Domain (RBD), S trimer (LakePharma; 46438 and 46328) and S N Terminal Domain [NTD; produced in house (78)]. All antigens corresponded to the SARS-CoV-2 Wuhan-Hu-1 strain.

### Luminex Multiplex Binding Antibody Assay

Bead-coupling and multiplex Luminex assays were performed as previously described, with modifications (79). Thirty µg of each S antigen and 15µg of NP antigen were covalently coupled to six million fluorescently coded carboxylated magnetic MagPlex beads (Luminex Corporation, Austin, TX). Samples were tested in triplicate at a dilution of 1:400; 1,200 antigen-conjugated beads for each antigen were used per well. Total IgG and IgM antibody responses were detected with 20µl of phycoerythrin (PE)-labeled secondary antibodies specific for total IgG and IgM (Southern BioTech; Birmingham, AL) at a final concentration of 3µg/ml. Prior to use, biotinylated proteins corresponding to Fc gamma receptors FcγR2a, FcγR2b, FcγR3a and FcγR3b (produced under the direction of Dr. James Peacock in the Duke Human Vaccine Institute Research Protein Production Facility which received funding support from the Collaboration for Aids Vaccine Research Bill and Melinda Gates Foundation (OPP1066832)) were mixed with a 1/4th molar ratio of Streptavidin-R-Phycoerthrin (SAPE; Prozyme) and incubated with rotation for 30 min at room temperature. One volume percent of 500µM free biotin (Thermo Fisher Scientific, Waltham, MA) was then added to block any free streptavidin binding sites and the SAPE-FcγRs were stored at 4°C and used within 24 hours. Binding to FcγRs was detected with 20µl of SAPE-FcγR2a, -FcγR2b, -FcγR3a and -FcγR3b at a final concentration of 1µg/ml.

Magnetic beads were acquired on a FlexMap 3D instrument (Luminex Corporation, Austin, TX) using the xPONENT software (version 4.2). A minimum of 100 beads per antigen and per well were collected. The output of the assay is the median fluorescence intensity (MFI) determined from the sampled beads. One single plate was run for each detection antibody and FcγR. Two pre-pandemic plasma samples from uninfected individuals were used as negative controls and two plasma samples from SARS-CoV-2 convalescent individuals were used as positive controls, each ran in triplicate at a 1:400 dilution. Each set of replicates was evaluated for outlier MFI values. The coefficient of variation (CV) of the triplicates was calculated; when the CV was higher than 20%, the replicate with the MFI signal furthest from the mean MFI was excluded. The mean MFI was then calculated for each sample.

### Data Analysis and Statistics

Multiple variable correlation analyses were performed using nonparametric Spearman correlation. Statistical differences between groups were determined using Wilcoxon matched pairs signed rank tests and Mann Whitney U tests.

## Data Availability Statement

The raw data supporting the conclusions of this article will be made available by the authors, without undue reservation.

## Ethics Statement

The studies involving human participants were reviewed and approved by Institutional Review Board at Walter Reed Army Institute of Research. The patients/participants provided their written informed consent to participate in this study.

## Author Contributions

LW, MZ, KP, JD, SP, and VP designed the studies. SP and VP provided project oversight. VD and SK contributed plasma and constructed SARS-CoV-2 S plasmids. MZ, SM, and EK developed and characterized the cell lines. MZ, EK, SM, and JH developed and performed SARS-CoV-2 infectivity, neutralization and FVE assays. MM, BB, SW-R, and MR performed SARS-CoV-2 Luminex antibody profiling. LW and MZ conducted data analysis. LW, MZ and VP wrote the manuscript. MM, VD, SW-R, KP, JD, SK, MR and SP reviewed and edited the manuscript. All authors contributed to the article and approved the submitted version.

## Funding

This work was supported by the U.S. Army Medical Research and Materiel Command under Contract No W81XWH-16-C-0337 between the Henry M. Jackson Foundation for the Advancement of Military Medicine, Inc. (HJF), and the U.S. Department of Defense (DoD), funded by the WRAIR Diagnostics and Countermeasure Branch. 

## Conflict of Interest

The authors declare that the research was conducted in the absence of any commercial or financial relationships that could be construed as a potential conflict of interest.

## Publisher’s Note

All claims expressed in this article are solely those of the authors and do not necessarily represent those of their affiliated organizations, or those of the publisher, the editors and the reviewers. Any product that may be evaluated in this article, or claim that may be made by its manufacturer, is not guaranteed or endorsed by the publisher.

## Supplementary Material

Supplementary Table 1 Correlation coefficients of binding antibody responses to Washington strain antigens (shown in rows) and Ig or Fc detectors (shown in columns) to plasma left) neutralization or right) FVE against the SARS-CoV-2 VOC and the Washington strain.

Supplementary Figure 1 Graphical summary of the route of viral entry (top) and the impact of antibody on viral entry (bottom) for **(A)** the HEK293_ FcγRIIa cell line, **(B)** the HEK293_ACE2 cell line and **(C)** primary cells, including monocytes and macrophages. The image was created using software available on BioRender.com.
