# Supplementary figures and images for: Evaluation of Antibody-Dependent Fc-Mediated Viral Entry, as Compared With Neutralization, in SARS-CoV-2 Infection
Source: Front Immunol. 2022 May 31;13:901217. doi: 10.3389/fimmu.2022.901217 (PMC9193970; doi:10.3389/fimmu.2022.901217)

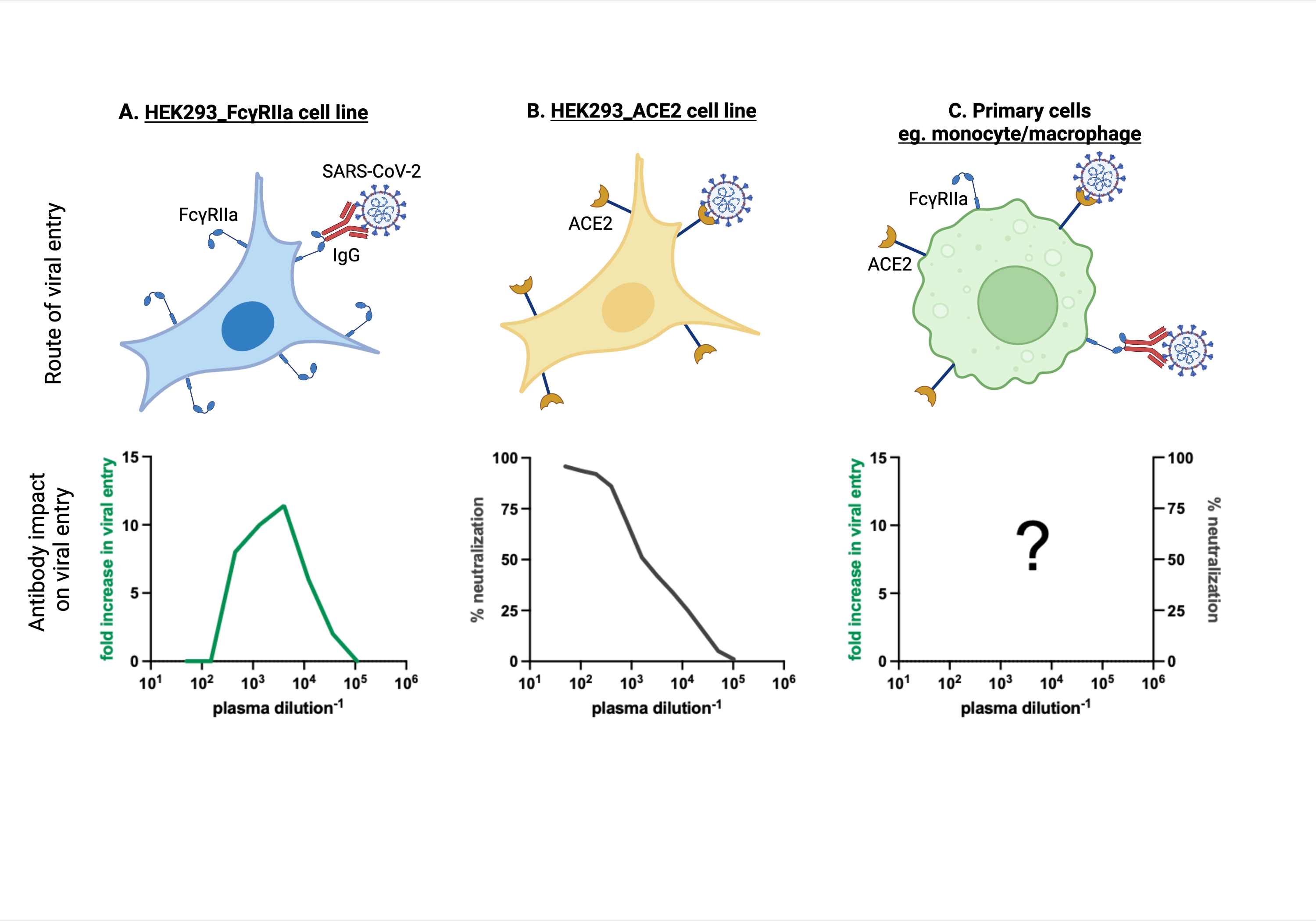

Supplement: Supplementary Figure 1 — Graphical summary of the route of viral entry (top) and the impact of antibody on viral entry (bottom) for (A) the HEK293_ FcγRIIa cell line, (B) the HEK293_ACE2 cell line and (C) primary cells, including monocytes and macrophages. The image was created using software available on BioRender.com. [file Image_1.jpeg]
